# Development and psychometric testing of a scale measuring caring behaviors for healthcare students and providers

**DOI:** 10.1080/10872981.2022.2066496

**Published:** 2022-04-17

**Authors:** Hung-Chang Liao, Cheng-Yi Huang, Ya-Huei Wang

**Affiliations:** a Department of Health Policy and Management, Chung Shan Medical University, Taichung, Taiwan; b Department of Medical Management, Chung Shan Medical University Hospital, Taichung, Taiwan; c School of Nursing, Chung Shan Medical University, Taichung, Taiwan; Department of Nursing, Chung Shan Medical University Hospital, Taichung, Taiwan; d Department of Applied Foreign Languages, Chung Shan Medical University, Taichung, Taiwan; e Department of Medical Education, Chung Shan Medical University Hospital, Taichung, Taiwan

**Keywords:** Caring behavior, compassion, healthcare students and providers, scale development, psychometric properties

## Abstract

This study intended to develop and assess the psychometric properties of a caring behavior scale on healthcare students and providers (CBS-HSP) in a Taiwanese population. After a literature review was conducted and an expert panel was consulted for item generation, 104 items for the CBS-HSP scale were derived on a nine-point Likert scale, with 9 indicating ‘extremely important’ and 1 indicating ‘extremely unimportant.’ A pilot study was then conducted with seven hundred forty-eight healthcare students and providers in Taiwan for further data analysis. The statistic software used in the study was SPSS for the exploratory factor analysis (EFA) and AMOS for the confirmatory factor analysis (CFA). Also, to examine the psychometric properties of the scale, internal consistency, convergent validities, discriminant validities, and model fit indices were calculated in the study. The EFA results derived 31 items in four factors, with 65.742% of the total variance explained: ‘support and attentiveness’ (11 items; 48.714% of the variance explained), ‘professional knowledge and skills’ (8 items; 8.226% of the variance explained), ‘gratifying needs and responsiveness’ (7 items; 5.236% of the variance explained), and ‘confidentiality and trust’ (5 items; 3.566% of the variance explained). The Cronbach’s alphas for the four subscales and the overall scale ranged from 0.894 to 0.964. The CFA results yielded the same 31 items, with the same four factors. The CFA results demonstrated good to excellent model fit in the χ2/df ratio (1.242), GFI (0.988), CFI (0.988), TFI (0.985), and RMSEA (0.031). The Cronbach’s alphas ranged between 0.866 and 0.971; the composite alphas ranged between 0.854 and 0.964. The convergent and discriminant validities also proved the stability of the CBS-HSP scale. The research results indicated that the developed CBS-HSP appeared to be a reliable instrument to measure healthcare students’ and providers’ caring behaviors.

## Introduction

Caring, an adjective related to compassion, has been defined as a sympathetic consciousness of others’ suffering, with a desire to alleviate it [1]. Caring, which is an emotional response to others’ pain and suffering and a desire to help [2,3], has been a vital component in the patient-and-healthcare-provider relationship in order to maintain high-quality healthcare and positive clinical and healthcare outcomes [4,5]. However, research has shown that patients have complained about healthcare providers’ poor attitudes and uncaring behaviors and have observed that healthcare providers do not take patients’ dignity and well-being into consideration [6,7]. Patients have also reflected that healthcare providers always distance themselves from patients and provide impersonal care [7]. Moreover, due to an overemphasis on the use of medical technology for clinical and healthcare practice, healthcare providers have paid more attention to checking the numbers and symbols displayed on the medical equipment, hence neglecting interactions with patients and patient families [8]. Focusing too much on medical technology, healthcare providers have gradually lost their awareness that the delivery of compassion and care is the core of the patient-and-healthcare-provider relationship [9].

Caring, which involves respect, honor, assurance, and so forth, has been regarded as the essence of medical care in that it is a humanistic behavior that gives holistic assistance to patients [10]. Caring behaviors involve with the actions and the concern of the well-being of patients, such as sensitivity, comfort, listening, honesty, putting patients first, etc. [11,12]. As Watson [13] says, caring behaviors involve more than words, actions, body languages; they also involve recognition, feeling, and thoughts to bring well-being to patients [14,15]. When a medical care system focuses too much on science and technology, the lack of caring behavior can hinder the simplification and routinization of medical care treatment and processes. Healthcare providers’ compassionate and caring behaviors are connected to their patients’ clinical and healthcare outcomes and, hence, their healthcare costs. Indeed, research has shown a positive association between compassionate patient-centered care and better clinical and healthcare outcomes [4,16]. Those receiving less care and compassion from healthcare providers reflected a lower quality of healthcare service and thus less healthcare satisfaction [17]. Therefore, in order to enhance the quality of healthcare, it is necessary to focus on compassionate, patient-centered care to improve healthcare outcomes [9,18]. Based on patients’ complaints about their healthcare providers’ attitudes and behaviors [6,7], in order to address the relationship between patients and healthcare providers, there is a need to develop strategies involving the transmission of care and humanistic qualities to healthcare students and providers because, when care is delivered in a humanistic way, it can lead to better medical and health care outcomes [19].

Although humanistic, caring behavior has been regarded as important to medical care practice, little is known about how to facilitate humanistic caring behaviors and attitudes in medical or healthcare education [19]. Moreover, though a variety of measures assess compassion and empathy from the perspective of healthcare providers [20–22], the caring behavior scales have been designed to measure patients’ intended caring behaviors. There are no caring behavior scales focused on measuring the caring behaviors across healthcare providers in the clinical and healthcare sectors [23]. Therefore, it is necessary to develop a caring behavior scale that pertains to healthcare providers; then, healthcare educators can realize their caring attitudes and behaviors and hence design teaching strategies, such as role modeling, programs, or on-the-job training, to help healthcare students or providers internalize humanistic caring behaviors and attitudes [24].

Thus, in order to help healthcare instructors measure healthcare students’ and providers’ caring behaviors and thereby design relevant learning strategies or activities to sharpen their humanistic caring behaviors, this study intended to develop and assess the psychometric properties of a caring behavior scale on healthcare students and providers in a Taiwanese population.

## Methodology

### Procedure and participants

To construct an instrument measuring the caring behavior of healthcare students and providers (CBS-HSP), while developing the scale, the researchers adopted the ten steps in three phases that were suggested by Slavec and Drnovšek [25]. Phase 1 regards the theoretical significance and construction, and includes three steps: specification of content domain (literature review, individual interviews, and focus group and stakeholder interviews), statement pool generation, and content validity assessment and confirmation. Phase 2 pertains to representativeness and adequacy of the data collection, and it includes four steps: scale development and evaluation, scale translation and back-translation, a pilot study, and data collection and sampling. Phase 3 involves statistical analysis and evidence of the construct, and it includes three steps: an examination of dimensionality, reliability, and construct validity.

In order to develop a valid and reliable CBS-HSP scale based on Taiwanese healthcare contexts, the interviews with students, stakeholders, and experts were conducted in Mandarin Chinese language and were used to provide the researchers a specific content domain of what caring behaviors perceived for further literature review for item generation. The interview results provided the researchers an initial specific content of the caring behaviors in the following areas: professional knowledge and skills, responding when needed, physical comfort, and positive communication. Also, after an extensive literature review, mainly from the four electronic databases: EBSCO, PubMed, ProQuest, and ScienceDirect, with the search terms of caring, caring behaviors, patient care, patient-centered care, compassion, communication, etc., the researchers collected 152 items in connection to healthcare students’ and providers’ caring behaviors. Then, the researchers convened panel discussion, with three experts specializing in psychometrics, healthcare education, and social sciences to examine the face validity and content validity of the scale items. The researchers let the 152 items be reviewed and rated by a panel of experts on a six-point (0–5) rating scale on how relevant the items measure the construct (0 = strongly irrelevant; 5 = strongly relevant). There might be any discrepancies between the rating results among experts; however, the items rated under 4 by any expert were deleted. Thus, the 152 items were reduced to 104 items, which were initially categorized into four categories as an a priori hypothetical model: ‘support and attentiveness,’ ‘professional knowledge and skills,’ ‘gratifying needs and responsiveness,’ and ‘confidentiality and trust.’ The CBS-HSP was on a nine-point Likert scale, in which 9 = extremely important, 8 = very important, 7 = moderately important, 6 = slightly important, 5 = neither important nor unimportant, 4 = slightly unimportant, 3 = moderately unimportant, 2 = very unimportant, and 1 = extremely unimportant. A higher score indicated a higher tendency toward caring behavior. Once the scale was initially developed, the researchers conducted a pilot study on seven hundred forty-eight healthcare students and providers in Taiwan for further the data analysis. The inclusion criteria for participants were those healthcare students or those with a healthcare educational background or expertise. The researchers used exploratory factor analysis (EFA), with a sample of 500 participants, to elicit underlying factor structure and used confirmatory factor analysis (CFA), with a sample of 248 participants, to further examine the derived factor structure.

The study received institutional review board approval (No. CS18216) from Chung Shang Medical University Hospital and was conducted according to the guidelines of the Research Ethics Framework of Society Institute in Taiwan [26].

### Data analysis

For the data analysis, the study used the Statistical Package for Social Sciences (SPSS; version 14.0) [27] for the exploratory factor analysis (EFA) to elicit the underlying structure. Also, the Kaiser-Meyer-Olkin test (KMO-test) [28] and Bartlett’s test of sphericity [29] were used to determine sampling adequacy. The study later used Analysis of Moment Structures (AMOS; version 24.0) [30] to perform confirmatory factor analysis (CFA) for further examination of the factor structure of the scale. In addition, Cronbach’s alphas and Pearson correlation coefficients were calculated to examine the internal consistency within a factor of test items and the correlation between any two factors.

Moreover, to examine the model’s goodness of fit, a number of model fit indices were used, including the χ2/df ratio [31], Tucker–Lewis Index (TLI) [32], comparative fit index (CFI) [33], goodness of fit index (GFI) [33], and root mean square error of approximation (RMSEA) [31]. In addition, the researchers tested the convergent validity of the CBS-HSP scale, using average variance extracted (AVE) values, and the discriminant validity, using the √AVE and correlation coefficients (*r*) between factors [34,35].

## Results

### EFA for the CBS-HP scale

Of the 500 valid participants, 361 participants (72.20%) were female and 139 participants were male (27.80%). Of these participants, 161 participants were healthcare professionals (32.20%) and 339 participants were healthcare students (67.80%), with 201 students studying in the college of medicine (59.29%), 55 students studying in the college of oral medicine (16.22%), 32 students studying in the college of health care and management (9.44%), and 51 students studying in the college of medical science and technology (15.05%). All these students had received service learning in healthcare settings as in medical institutions, social welfare communities, public health bureaus, or health centers.

#### Bartlett’s test of sphericity and KMO measure

**Figure 1.**
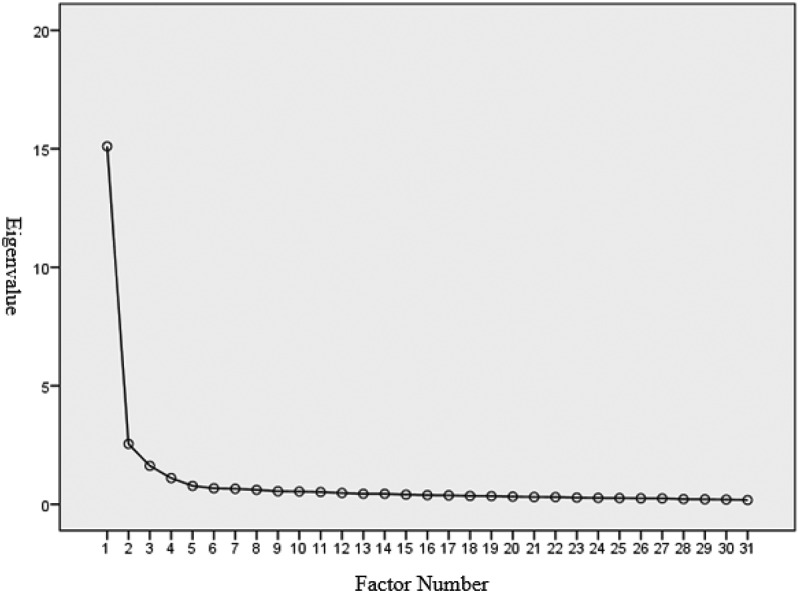
Scree plot for factor analysis of the CBS-HSP.

The study used Bartlett’s test of sphericity and KMO measure to demonstrate the sample size adequacy and to determine the worthiness of factor analysis. The KMO value of 0.966, higher than the threshold value of 0.6, has been proven to be extremely good [28]. Bartlett’s [29] test of sphericity was less than 0.05 (Approx. = 11,258.982; degrees of freedom = 465, *p* value = 0.000 < 0.05). The results of Bartlett’s test and the KMO measure proved the appropriateness of the sample size for the EFA. The scree-plot test (Figure 1) for factor analysis of the CBS-HSP also suggests the four-factor solution.

#### EFA and Principal Component Analysis (PCA)

EFA was adopted to test the construct validity and the internal consistency of the scale, using PCA and a promax rotation. Also, the eigenvalues should be greater than 1.0. based on Hair et al.’s criteria on factor structure [34], and the factor loadings should be higher than 0.5; those greater than 0.70 are considered good to explain a variable. Hence, in order for the CBS-HSP scale to be a well-defined factor structure, while selecting the items, the researchers retained the items whose factor loadings were greater than 0.70 (in absolute value) for the relevant factor and less than 0.70 for the nonrelevant factors. There was no double loading between any two items.

After conducting the EFA and PCA, four factors and 31 items were derived: ‘support and attentiveness,’ ‘professional knowledge and skills,’ ‘gratifying needs and responsiveness,’ and ‘confidentiality and trust.’ These four factors explained 65.742% of the variance (see Table 1). Factor 1, with 11 items regarding ‘support and attentiveness,’ explained 48.714% of the variance. A sample item for factor 1 is ‘As a healthcare professional, I would accompany patients in order to give them spiritual support.’ Factor 2, with 8 items on ‘professional knowledge and skills,’ explained 8.226% of the variance. A sample item for factor 2 is ‘As a healthcare professional, I would monitor and track the progress of patients’ course of disease to examine the effectiveness of the healthcare services.’ Factor 3, with 7 items regarding ‘gratifying needs and responsiveness,’ explained 5.236% of the variance. A sample item for factor 3 is ‘As a healthcare professional, when something goes wrong with patients’ healthcare, I would immediately report the situation to senior staff or relative professionals.’ Factor 4, with 5 items regarding ‘confidentiality and trust,’ explained 3.566% of the variance. A sample item for factor 4 is ‘As a healthcare professional, I would keep patients’ condition or personal privacy confidential.’ All of the eigenvalues for the four factors from the PCA were larger than one: 15.101, 2.550, 1.623, and 1.106 (see Table 1). These results support the multidimensionality of the CBS-HSP scale.

**Table 1.** Rotated factor loadings and Cronbach’s alphas for the CBS-HSP

Item	Factor 1: Support & Attentiveness	Factor 2: Professional Knowledge & Skills	Factor 3: Gratifying Needs & Responsiveness	Factor 4: Confidentiality & Trust
Factor 1: α = 0.934				
60	0.802			
90	0.798			
81	0.794			
88	0.792			
93	0.791			
104	0.780			
103	0.760			
64	0.757			
63	0.756			
58	0.756			
73	0.734			
Factor 2: α = 0.926				
13		0.839		
18		0.821		
5		0.820		
10		0.819		
17		0.817		
20		0.810		
8		0.795		
1		0.759		
Factor 3: α = 0.910				
51			0.851	
50			0.831	
43			0.816	
44			0.795	
42			0.791	
57			0.725	
68			0.717	
Factor 4: α = 0.894				
27				0.878
26				0.844
24				0.832
14				0.832
25				0.797
Eigenvalue	15.101	2.550	1.623	1.106
% of variance	48.714	8.226	5.236	3.566

Overall = 0.964; total variance explained is 65.742%

#### Validity and reliability analysis of the EFA model for the CBS-HSP scale

The scale was initially developed in English, then were translated forward into Mandarin Chinese, and after that, translated backward into English. Both the Mandarin Chinese version and English version were reviewed by two bilingual teachers, with minor revisions made to confirm their content validity. To examine the internal consistency, the researchers calculated the Cronbach’s alpha [36,37] for each factor and the overall scale to measure the coherence of the constructs.

Generally, the minimum criteria for an acceptable Cronbach’s alpha is 0.70 [37]. The statistic results showed that the Cronbach’s alpha values for the four subscales were 0.934, 0.926, 0.910, and 0.894 in ‘support and attentiveness,’ ‘professional knowledge and skills,’ ‘gratifying needs and responsiveness,’ and ‘confidentiality and trust,’ respectively; the Cronbach’s alpha for the entire CBS-HSP scale was 0.964 (see Table 1).

#### Scale item descriptions, item mean scores, and standard deviation

The CBS-HSP scale item descriptions, item mean scores, and standard deviations are shown as in Table 2.

**Table 2.** Scale item statements and descriptive statistics on the CBS-HSP scale

Scale Item Descriptions	Mean	*S.D.*
Factor 1: Support & Attentiveness	85.984	10.530
60. As a healthcare professional, no matter what happens, I would always give priority to patients.	7.83	1.170
90. As a healthcare professional, when talking to patients, I would look at them and smile sincerely.	7.75	1.293
81. As a healthcare professional, I would accompany patients in order to give them spiritual support.	7.99	1.157
88. As a healthcare professional, when patients need support, I would try to use words and actions to help them go through difficulties, for instance, accompanying patients and letting them know that I would be with them during their difficulties.	7.92	1.110
93. As a healthcare professional, I believe that a friendly relationship with patients would help them recover.	7.78	1.324
104. As a healthcare professional, I would put myself in the patients’ position to experience their pain or reactions.	7.80	1.239
103. As a healthcare professional, I would be able to understand how patients feel.	7.74	1.262
64. As a healthcare professional, I would express my concern to patients.	7.72	1.304
63. As a healthcare professional, I would praise patients to confirm their efforts. For example, I would tell patients, ‘You did a good job.’	7.88	1.250
58. As a healthcare professional, I would pay attention to patients’ complaints, no matter what the complaint is.	7.78	1.223
73. As a healthcare professional, I would try to help patients use their belief to support themselves as they go through the suffering of their disease.	7.79	1.231
Factor 2: Professional Knowledge & Skills	62.432	8.283
13. As a healthcare professional, I would try my best to use my professional knowledge, technology, and experience in medical, nursing, or healthcare practice to alleviate the symptoms of patients.	7.99	1.204
18. As a healthcare professional, I would monitor the progress of patients’ course of disease to examine the effectiveness of healthcare services.	7.85	1.281
5. As a healthcare professional, I would try my best to confirm that the patients’ healthcare plan is as they requested and meets its expected goals.	7.64	1.254
10. As a healthcare professional, I would try my best to assist my patients in making appropriate healthcare decisions.	7.69	1.287
17. As a healthcare professional, I would try my best to obtain patients’ healthcare history to create their healthcare plan.	7.79	1.250
20. As a healthcare professional, my routine professional healthcare service, combined with my relevant knowledge and experience, would help me provide professional service without difficulty.	7.87	1.228
8. As a healthcare professional, I would try my best to provide patients’ relevant healthcare information to healthcare teams in order to maintain the continuity of healthcare service.	7.70	1.330
1. As a healthcare professional, I would be able to identify the signs that patients’ conditions are beginning to deteriorate.	7.91	1.359
Factor 3: Gratifying Needs & Responsiveness	56.161	6.314
51. As a healthcare professional, I would try my best to provide patients and their families with relevant healthcare information regarding the patients’ disease in order to help them evaluate what kind of healthcare service they need.	8.04	1.098
50. As a healthcare professional, after helping patients complete their general physical examination (such as blood pressure) or physiological examination (such as electrocardiogram), I would try to actively inform them of the examination results or when they have to return to the hospital to see the examination report.	8.06	1.194
43. As a healthcare professional, when something goes wrong with patients’ healthcare, I would immediately report the situation to senior staff or relative professionals.	7.99	1.137
44. As a healthcare professional, when patients are uncomfortable or in pain, I would immediately try to help them alleviate their pain or discomfort.	7.92	1.136
42. As a healthcare professional, when patients are in need of being taken care of, I would try my best to meet their needs promptly.	7.92	1.118
57. As a healthcare professional, I would tell patients about their relevant examination, treatment, or healthcare service plans in order to let them be mentally prepared.	8.12	1.080
68. As a healthcare professional, I would be able to understand the needs of patients and provide appropriate healthcare services.	8.12	1.065
Factor 4: Confidentiality & Trust	41.414	4.484
27. As a healthcare professional, I would keep patients’ condition or personal privacy confidential.	8.39	1.000
26. As a healthcare professional, I would try to avoid unnecessary exposure of patients’ bodies during examination, treatments, or healthcare service by, for example, drawing curtains or covering patients’ bodies properly.	8.30	1.072
24. As a healthcare professional, I would treat patients as independent individuals.	8.27	1.066
14.As a healthcare professional, while making any healthcare decision, I think the opinions of patients and their families should always be taken into consideration.	8.35	1.013
25. As a healthcare professional, I think the relationship between patients and me should be based on mutual trust.	8.10	1.190

*S.D*. = Standard Deviation

### CFA for the CBS-HSP scale

After the use of EFA, the 104 items in the CBS-HSP scale were reduced to 31 items in four factors, with well-defined factor structure and internal consistency. To further confirm the factor structure of the EFA-derived scale, the researchers conducted CFA, using AMOS [30]. Of the 248 valid participants, 182 participants (73.39%) were female and 66 participants were male (26.61%). Of these participants, 87 participants were healthcare professionals (35.08%) and 161 participants were healthcare students (64.92%), with 76 students studying in the college of medicine (47.21%), 28 students studying in the college of oral medicine (17.39%), 19 students studying in the college of health care and management (11.80%), and 38 students studying in the college of medical science and technology (23.60%).

**Figure 2.**
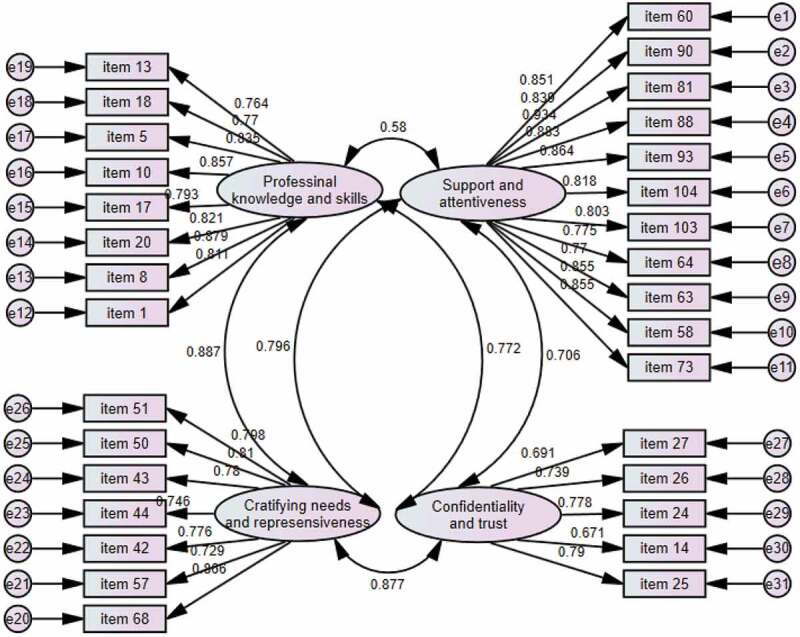
CFA for the CBS-HSP scale.

The CFA yielded the same four factors and 31 items without deleting any items: ‘support and attentiveness’ (11 items; factor loadings: 0.770–0.934), ‘professional knowledge and skills’ (8 items; factor loadings: 0.764–0.879), ‘gratifying needs and responsiveness’ (7 items; factor loadings: 0.729–0.866), and ‘confidentiality and trust’ (5 items; factor loadings: 0.671–0.790). The four-factor CFA for the 31 items is shown in Figure 2 and in appendix.

#### Model fit of the CBS-HSP scale

The study used a variety of model fit indices – the χ2/df ratio, GFI, CFI, TLI, and RMSEA – to look into how well the derived CBS-HSP fit the data (see Table 3). A χ2/df ratio value below 5 is regarded as a good model fit [31,38]. A GFI higher than 0.90 is regarded as good [33]. A CFI greater than or equal to 0.90 is regarded as acceptable, and one with an index greater than 0.95 is considered excellent [32,39]. A TLI greater than or equal to 0.90 is considered acceptable, and one with an index value greater than 0.95 is considered excellent [32,40,41]. An RMSEA index less than 0.08 is regarded as acceptable, and one with an index below 0.05 is considered excellent [33]. In the EFA-derived CBS-HSP, the χ2/df ratio is 2.720, GFI is 0.774, CFI is 0.900, TLI is 0.891, RMSEA is 0.083, and CI for RMSEA is [0.078, 0.089]. In the CFA-derived scale, the χ2/df ratio is 1.242, GFI is 0.988, CFI is 0.988, TLI is 0.985, RMSEA is 0.031, and CI for RMSEA is [0.021, 0.040].

**Table 3.** Model fit for the EFA-derived and CFA-derived CBS-HSP

	$\chi^{2}/df$	GFI	CFI	TLI	RMSEA	90% RMSEA CI
EFA-derived Scale	2.720	0.774	0.900	0.891	0.083	[0.078, 0.089]
CFA-derived Scale	1.242	0.988	0.988	0.985	0.031	[0.021, 0.040]

Note: *N* = 248; χ2 = Chi-square; df = degree of freedom; GFI: goodness of fit index; CFI = comparative fit index; TLI = Tucker Lewis index; RMSEA = root mean square error of approximation; CI = Confidential Index.

#### Reliability

The Cronbach’s alphas for the four subscales were 0.965, 0.944, 0.923, and 0.866, respectively, and the Cronbach’s alpha for the entire questionnaire was 0.971. The researchers further used the composite reliabilities to verify the CBS-HSP scale’s stability and internal consistency. The derived composite reliability coefficients were 0.964, 0.941, 0.919, and 0.854 (see Table 4).

**Table 4.** AVE (average variance extracted), Cronbach’s alphas, and composite alphas

AVE & Composite Alpha Factor	AVE	Cronbach’s Alpha – EFA-Derived Scale	Cronbach’s Alpha – CFA-Derived Scale	Composite Alpha – CFA-Derived Scale
1. Support and attentiveness	0.709	0.934	0.965	0.964
2. Professional knowledge and skills	0.668	0.926	0.944	0.941
3. Gratifying needs and responsiveness	0.620	0.910	0.923	0.919
4. Confidentiality and trust	0.541	0.894	0.866	0.854

#### Convergent validity

The researchers analyzed the convergent validity, an index of internal consistency, to examine whether the factor items were related to the respective factor by calculating the AVE (average variance extracted) of each factor and comparing it to its composite reliability with other factors [34,42]. Convergent validity is verified if the AVE of the factor is above 0.50 and smaller than its corresponding composite reliability. In fact, the AVE values of the four subscales are above 0.50 (see Table 4): 0.709 (‘support and attentiveness’), 0.668 (‘professional knowledge and skills’), 0.620 (‘gratifying needs and responsiveness’), and 0.541 (‘confidentiality and trust’). The corresponding composite reliabilities are 0.964, 0.941, 0.919, and 0.854, respectively.

#### Discriminant validity

To examine the CBS-HSP’s discriminant validity, the researchers calculated the square root values of the AVE (√AVE) and compared the √AVE values to the corresponding Pearson’s correlation coefficient (as Pearson’s *r*) between factors [35]. Discriminant validity is achieved when the factor’s √AVE value is higher than its correlations with other factors [34,42]. As shown in Table 5, the discriminant validity is demonstrated between the factors of ‘support and attentiveness’ and ‘professional knowledge and skills’ (√AVE = 0.842 and 0.817, respectively; *r* = 0.548), between the factors of ‘support and attentiveness’ and ‘gratifying needs and responsiveness’ (√AVE = 0.842 and 0.787, respectively; *r* = 0.720), between the factors of ‘support and attentiveness’ and ‘confidentiality and trust’ (√AVE = 0.842 and 0.735, respectively; *r* = 0.603), between the factors of ‘professional knowledge and skills’ and ‘gratifying needs and responsiveness’ (√AVE = 0.817 and 0.787, respectively; *r* = 0.817), and between the factors of ‘professional knowledge and skills’ and ‘confidentiality and trust’ (√AVE = 0.817 and 0.735, respectively; *r* = 0.653). There is a marginal discriminant validity between the factors of ‘gratifying needs and responsiveness’ and ‘confidentiality and trust’ (√AVE = 0.787 and 0.735, respectively; *r* = 0.773). The discriminant validities and Pearson correlation coefficients are as Table 5.

**Table 5.** Discriminant validities of the CFA-derived CBS-HSP scale

Factor	1	2	3	4
1. Support and attentiveness	**0.842**			
2. Professional knowledge and skills	0.548**	**0.817**		
3. Gratifying needs and responsiveness	0.720**	0.817**	**0.787**	
4. Confidentiality and trust	0.603**	0.653**	0.773**	**0.735**

The values shown in bold are the square root of AVE (√AVE)

The values shown with ** are the Pearson correlation coefficients.

**p < 0.001

## Discussion

This study aimed to develop an instrument to measure healthcare students’ and providers’ caring behaviors in Taiwanese healthcare cultural contexts. After the literature review, experts screened out inappropriate items and categorized the items into four categories as an a priori hypothetical model: ‘support and attentiveness,’ ‘professional knowledge and skills,’ ‘gratifying needs and responsiveness,’ and ‘confidentiality and trust.’

According to Bartlett [43], the spherical value should be statistically significant with a *p* value less than 0.05. Based on Kaiser [28], a KMO value above 0.60 is regarded as acceptable, a value in the range of 0.70 to 0.80 is fair, a value between 0.80 and 0.90 is good, and a value above 0.90 is deemed perfect [44,45]. In the EFA-derived CBS-HSP, with the Bartlett spherical value less than 0.01 and the perfect KMO value of 0.966, the CBS-HSP has demonstrated its adequate sample size and the worthiness of factor analysis. The scree plot in factor extraction also indicated that the four-factor solution was the best model for following analysis.

Although the experts reached a consensus regarding the content of the scale items and categories, it may have been unnecessary to perform the EFA in the study. However, to examine the a priori hypothetical model, being conservative, the researchers first conducted an EFA on the dataset to examine the a priori hypothetical model and then used the CFA to verify whether the dataset was suitable for the model [46]. After the application of EFA, 31 items were derived in four factors, explaining 65.742% of the variance: ‘support and attentiveness’ (11 items), ‘professional knowledge and skills’ (8 items), ‘gratifying needs and responsiveness’ (7 items), and ‘confidentiality and trust’ (5 items). The factor loadings in the EFA results ranged from 0.717 to 0.878, all greater than Hair et al.’s suggested factor loading, thus demonstrating evidence for a well-defined factor structure of the scale [34].

The subscale means showed that the respondents scored the highest on the ‘confidentiality and trust’ subscale (mean = 8.283; 41.414 ÷ 5 = 8.283), followed by ‘gratifying needs and responsiveness’ (mean = 8.023) and ‘support and attentiveness’ (mean = 7.817). The participants scored the lowest on the ‘professional knowledge and skills’ (mean = 7.804). The subscale means showed that these respondents think that the priorities of caring behavior should focus on keeping a patient’s condition or personal information confidential; they should also avoid unnecessarily exposing patients’ bodies during examination, treatments, or healthcare service. Next in priority is to gratify needs and respond to patients. For instance, when patients need to be taken care of, healthcare professionals should try their best to meet those needs promptly. When patients are uncomfortable or ill, healthcare professionals should immediately take relevant measures to alleviate their patients’ discomfort. Compared with caring behavior in connection to ‘confidentiality and trust,’ ‘gratifying needs and responsiveness,’ and ‘support and attentiveness,’ the respondents think that professional knowledge and skills are relatively less important for caring behavior. The researchers applied CFA to further examine the factorial validity, which yielded the same 31 items, with the factor loadings varying between 0.934 and 0.671; this was greater than Hair et al.’s [34] suggested criteria for factor loading.

All fit indices in the CFA-derived CBS-HSP scale met the benchmarks of the model fit [31–43; 39–42], indicating an acceptable or excellent model fit. Moreover, compared with the good model fit indices in the EFA-derived CBS-HSP, the indices in the CFA-derived CBS-HSP have shown a better model fit, with an increase of 0.214, 0.088, and 0.094 in GFI, CFI, and TLI, respectively, a decrease of 0.052 in RMSEA, and a decrease of 1.478 in the χ2/df ratio value. In addition, the Cronbach’s alphas and composite reliabilities also meet Hair et al.’s criterion [34]. As for the internal consistency, the Cronbach’s alphas and the composite reliabilities ranged between 0.854 and 0.971, meeting Hair et al.’s^29^ suggested cutoff value of 0.70. Thus, with excellent reliabilities, the CBS-HSP scale can be used to assess participants’ caring behavior. The convergent validities were also demonstrated. Based on Hair et al. and Malhorta [34,42], convergent validity is demonstrated when the AVE of the factor is greater than 0.50 and less than its corresponding composite reliability. In the study, all of the AVE values of ‘support and attentiveness,’ ‘professional knowledge and skills,’ ‘gratifying needs and responsiveness,’ and ‘confidentiality and trust’ are greater than the benchmark criteria of 0.50 and less than the corresponding composite values [34,42]. The discriminant validities were also demonstrated, meeting Hair et al.’s [34] and Fornell-Larcker’s criteria [47]. However, criterion validity was not taken into consideration in the study because there have been no existing scales measuring healthcare students’ and providers’ caring behaviors. Also, considering that foreign scales derived from different cultures may not be an appropriate benchmark test used among Taiwanese participants [48], the researchers excluded the criterion validity in the study. In future study, the researchers would manage to find a benchmark test with intercultural validity and reliability and use that test to examine the criterion validity of the CBS-HSP scale.

With the factorial structure and psychometric properties being scrutinized and validated, several limitations of the study should be pointed out. This caring behavior scale was grounded in a Taiwanese healthcare cultural context, with the healthcare students and providers as samples. Hence, for those intending to use the scale to measure participants’ caring behavior, they should take sample similarity into consideration. In addition, the CBS-HSP was constructed based on the sample of healthcare students and providers; hence, it may be inappropriate to let patients and patient families complete the scale to realize their expected caring behavior. Future studies may also attempt to develop a caring behavior scale using patients and patient families as the sample to realize what caring behaviors they are expecting. Future studies may also compare the differences in the expected caring behaviors between healthcare students/providers and patients/patient families; with the collected data, researchers may determine a means to facilitate a positive healthcare relationship between patients and providers.

## Conclusion

Given that healthcare providers’ caring and compassion are prerequisites for quality healthcare, this study aimed to develop an instrument measuring healthcare students’ and providers’ caring behaviors. The research results proved that the developed CBS-HSP appears to be a reliable tool to measure healthcare students’ and providers’ caring behaviors. The assistance of the CBS-HSP scale may help healthcare instructors measure students’ and professionals’ caring behaviors and hence design relevant learning strategies or activities to sharpen their humanistic caring behaviors.

## Appendix. The factors and items of the CFA-derived CBS-HSP scale

Factor 1: Support & Attentiveness

1. As a healthcare professional, no matter what happens, I would always give priority to patients.
2. As a healthcare professional, when talking to patients, I would look at them and smile sincerely.
3. As a healthcare professional, I would accompany patients in order to give them spiritual support.
4. As a healthcare professional, when patients need support, I would try to use words and actions to help them go through difficulties, for instance, accompanying patients and letting them know that I would be with them during their difficulties.
5. As a healthcare professional, I believe that a friendly relationship with patients would help them recover.
6. As a healthcare professional, I would put myself in the patients’ position to experience their pain or reactions.
7. As a healthcare professional, I would be able to understand how patients feel.
8. As a healthcare professional, I would express my concern to patients.
9. As a healthcare professional, I would praise patients to confirm their efforts. For example, I would tell patients, ‘You did a good job.’
10. As a healthcare professional, I would pay attention to patients’ complaints, no matter what the complaint is.
11. As a healthcare professional, I would try to help patients use their belief to support themselves as they go through the suffering of their disease.

Factor 2: Professional Knowledge & Skills

1. As a healthcare professional, I would try my best to use my professional knowledge, technology, and experience in medical, nursing, or healthcare practice to alleviate the symptoms of patients.
2. As a healthcare professional, I would monitor the progress of patients’ course of disease to examine the effectiveness of healthcare services.
3. As a healthcare professional, I would try my best to confirm that the patients’ healthcare plan is as they requested and meets its expected goals.
4. As a healthcare professional, I would try my best to assist my patients in making appropriate healthcare decisions.
5. As a healthcare professional, I would try my best to obtain patients’ healthcare history to create their healthcare plan.
6. As a healthcare professional, my routine professional healthcare service, combined with my relevant knowledge and experience, would help me provide professional service without difficulty.
7. As a healthcare professional, I would try my best to provide patients’ relevant healthcare information to healthcare teams in order to maintain the continuity of healthcare service.
8. As a healthcare professional, I would be able to identify the signs that patients’ conditions are beginning to deteriorate.

Factor 3: Gratifying Needs & Responsiveness

1. As a healthcare professional, I would try my best to provide patients and their families with relevant healthcare information regarding the patients’ disease in order to help them evaluate what kind of healthcare service they need.
2. As a healthcare professional, after helping patients complete their general physical examination (such as blood pressure) or physiological examination (such as electrocardiogram), I would try to actively inform them of the examination results or when they have to return to the hospital to see the examination report.
3. As a healthcare professional, when something goes wrong with patients’ healthcare, I would immediately report the situation to senior staff or relative professionals.
4. As a healthcare professional, when patients are uncomfortable or in pain, I would immediately try to help them alleviate their pain or discomfort.
5. As a healthcare professional, when patients are in need of being taken care of, I would try my best to meet their needs promptly.
6. As a healthcare professional, I would tell patients about their relevant examination, treatment, or healthcare service plans in order to let them be mentally prepared.
7. As a healthcare professional, I would be able to understand the needs of patients and provide appropriate healthcare services.

Factor 4: Confidentiality & Trust

1. As a healthcare professional, I would keep patients’ condition or personal privacy confidential.
2. As a healthcare professional, I would try to avoid unnecessary exposure of patients’ bodies during examination, treatments, or healthcare service by, for example, drawing curtains or covering patients’ bodies properly.
3. As a healthcare professional, I would treat patients as independent individuals.
4. As a healthcare professional, while making any healthcare decision, I think the opinions of patients and their families should always be taken into consideration.
5. As a healthcare professional, I think the relationship between patients and me should be based on mutual trust.

## References

[1] Fan VY, Lin SC. It is time to include compassion in medical training. Acad Med. 2013;88:11. 10.1097/ACM.0b013e3182754024 23267223

[2] Sinclair S, Norris JM, McConnell SJ, et al. Compassion: a scoping review of the healthcare literature. BMC Palliat Care. 2016;15:6. 26786417 10.1186/s12904-016-0080-0 PMC4717626

[3] Singer T, Klimecki OM. Empathy and compassion. Curr Biol. 2014;24:R875–R878. 25247366 10.1016/j.cub.2014.06.054

[4] Lown BA, Rosen J, Marttila J. An agenda for improving compassionate care: a survey shows about half of patients say such care is missing. Health Affairs (Millwood). 2011;30:1772–11. 10.1377/hlthaff.2011.0539 21900669

[5] Trzeciak S, Roberts BW, Mazzarelli AJ. Compassionomics: hypothesis and experimental approach. Med Hypotheses. 2017;107:92–97. 28915973 10.1016/j.mehy.2017.08.015

[6] Santo-Novak DA. Older adults’ descriptions of their role expectations of nursing. J Gerontol Nurs. 1997;23:32–40. 10.3928/0098-9134-19970101-11 9136368

[7] Hogg R, Hanley J, Smith P. Learning lessons from the analysis of patient complaints relating to staff attitudes, behaviour and communication, using the concept of emotional labour. J Clin Nurs. 2018;27:e1004–12. 29052343 10.1111/jocn.14121

[8] Watson J. Caring theory as an ethical guide to administrative and clinical practices. Nurs Adm Q. 2006;30:48–55. 16449884 10.1097/00006216-200601000-00008

[9] Bertakis KD, Azari R. Patient-centered care is associated with decreased health care utilization. J Am Board Fam Med. 2011;24:229–239. 21551394 10.3122/jabfm.2011.03.100170

[10] Tonges M, Ray J. Translating caring theory into practice: the Carolina care model. J Nurs Adm. 2011;4:374–381. 10.1097/NNA.0b013e31822a732c 21881444

[11] Larson PJ. Cancer nurses’ perceptions of caring. Cancer Nurs. 1986;9:86–91. 3635439

[12] Larson PJ, Ferketich SL. Patients’ satisfaction with nurses caring during hospitalization. West J Nurs Res. 1993;15:690Y707. 8284928 10.1177/019394599301500603

[13] Watson J. Nursing: the philosophy and science of caring. Boulder/Colorado: University Press of Colorado; 2008.

[14] Latham C. Predictors of patient outcomes following interactions with nurses. West J Nurs Res. 1996;18:548–564. 8918207 10.1177/019394599601800506

[15] Wolf ZR, Miller PA, Devine M. Relationship between nurse caring and patient satisfaction in patients undergoing invasive cardiac procedures. MedSurg Nursing J. 2003;12:391–396. 14725151

[16] Neumann M, Wirtz M, Bollschweiler E, et al. Determinants and patient-reported long-term outcomes of physician empathy in oncology: a structural equation modelling approach. Patient Educ Couns. 2007;69:63–75. 17851016 10.1016/j.pec.2007.07.003

[17] West CP, Huschka MM, Novotny PJ, et al. Association of perceived medical errors with resident distress and empathy: a prospective longitudinal study. JAMA. 2006;296:1071–1078. 16954486 10.1001/jama.296.9.1071

[18] Stewart M, Brown JB, Donner A, et al. The impact of patient-centered care on outcomes. J Fam Pract. 2000;49:796–804. 11032203

[19] Weissmann PF, Branch WT, Gracey CF, et al. Role modeling humanistic behavior: learning bedside manner from the experts. Acad Med. 2006;82:661–667. 10.1097/01.ACM.0000232423.81299.fe 16799294

[20] Burnell L, Agan DL. Compassionate care: can it be defined and measured? the development of the compassionate care assessment tool. Int J Caring Sci. 2013;6:180–187.

[21] Lown BA, Muncer SJ, Chadwick R. Can compassionate healthcare be measured? the Schwartz center compassionate care scale. Patient Educ Couns. 2015;98:1005–1010. 25921380 10.1016/j.pec.2015.03.019

[22] Mercer SW, Maxwell M, Heaney D, et al. The Consultation and Relational Empathy (CARE) measure: development and preliminary validation and reliability of an empathy-based consultation process measure. Fam Pract. 2004;21:699–705. 15528286 10.1093/fampra/cmh621

[23] Roberts BW, Roberts MB, Yao J, et al. Development and validation of a tool to measure patient assessment of clinical compassion. JAMA Network Open. 2019;2:e193976. 31099870 10.1001/jamanetworkopen.2019.3976 PMC6537812

[24] Lee-Hsieh J, Kuo CL, Tsai YH. An action research on the development of a caring curriculum in Taiwan. J Nurs Educ. 2004;43:391–400. 15478691 10.3928/01484834-20040901-03

[25] Slavec A, Drnovšek M. Perspective on scale development in entrepreneurship research. Econ Bus Rev. 2012;14:39–62.

[26] TSA. Research ethics framework of society institute in Taiwan. Available online: http://proj3.sinica.edu.tw/~tsa/modules/tadnews/index.php?nsn=43 (accessed on 2016 Nov 26).

[27] IBM Corp. IBM SPSS statistics for windows, version 24.0. Armonk/NY: IBM Corp; 2016.

[28] Kaiser HF. An index of factorial simplicity. Psychometrika. 1974;39:31–36.

[29] Bartlett MS. A further note on tests of significance in factor analysis. Br J Psychol. 1951;4:1–2.

[30] Arbuckle JL. IBM SPSS Amos 24 User’s Guide. New York/NY: IBM; 2015.

[31] Hooper D, Coughlan J, Mullen M. Structural equation modelling: guidelines for determining model fit. Electron J Business Res Methods. 2008;6:53–60.

[32] Bentler PM. Comparative fit indexes in structural models. Psychol Bull. 1990;107:238–246. 2320703 10.1037/0033-2909.107.2.238

[33] Hu L, Bentler PM. Cutoff criteria for fit indexes in covariance structure analysis: conventional criteria versus new alternatives. Struct Equ Modeling. 1999;6:1–55.

[34] Hair J, Black W, Babin B, et al. Multivariate data analysis. 7th ed. Upper Saddle River/NJ: Prentice-Hall; 2010.

[35] Gefen D, Straub D, Boudreau MC. Structural equation modeling and regression: guidelines for research practice. Commun AIS. 2000;4:1–78.

[36] Cronbach LJ. Coefficient alpha and the internal structure of tests. Psychometrika. 1951;16(3):297–334.

[37] Nunnally JC. Psychometric Theory. 2nd ed. New York/NY: McGraw-Hill; 1978.

[38] Tabachnick BG, Fidell LS. Using multivariate statistics. 5th ed. New York/NY/USD: Allyn and Bacon; 2007.

[39] Sharma S, Mukherjee S, Kumar A, et al. A simulation study to investigate the use of cutoff values for assessing model fit in covariance structure models. J Bus Res. 2005;58:935–943.

[40] Ma Z. Application of structural equation modeling to evaluate customer satisfaction in the China internet bank sector. In *Proceedings of the International Conference on Instrumentation, Measurement, Circuits and Systems* (ICIMCS 2011), Hong Kong, China, 2011 Dec 12–13; ASME International, 2011; pp. 975–978.

[41] Schermelleh-Engel K, Moosbrugger H. Evaluating the fit of structural equation models: tests of significance and descriptive goodness-of-fit measures. Methods Psychol Res Online. 2003;8:23–74.

[42] Malhotra NK. Pesquisa de Marketing: uma Orientação Aplicada. 6th ed. São Paulo/Brazil: Bookman; 2011.

[43] Bartlett MS. A note on the multiplying factors for various chi-square approximations. J R Stat Soc. 1954;16:296–298.

[44] Cinar D, Yava A. Validity and reliability of functional assessment of chronic illness treatment-fatigue scale in Turkish patients with type 2 diabetes. Endocrinología, Diabetes y Nutrición. 2018;65:409–417. 10.1016/j.endinu.2018.01.010 29685730

[45] Yalçiner N, Türkmen SN, Irmak H, et al. The validity and reliability of the Turkish Form of Recovery Process Inventory. Anatolian J Psychiatry. 2019;20:32–40.

[46] Menezes MS, Gusmão MM, Santana RM, et al. Translation, transcultural adaptation, and validation of the role-modeling cost-conscious behaviors scale. BMC Med Educ. 2019;19:151. 31096964 10.1186/s12909-019-1587-x PMC6524215

[47] Fornell C, Larcker DF. Evaluating structural equation models with unobservable variables and measurement error. J Marketing Res. 1981;18:39.

[48] Stevens JP. Applied multivariate statistics for the social sciences. 5th ed. New York/NY: Routledge; 2009.

